# Dual targeting of *CD155*/*TIGIT* and *PD-L1*/*PD-1* immune checkpoints potentiates NK cell-mediated cytotoxicity in medulloblastoma

**DOI:** 10.1093/noajnl/vdaf099

**Published:** 2025-05-18

**Authors:** Matthijs Monnikhof, Michael Y Schakelaar, Chris Meulenbroeks, Matthias Quist, Alicia Perzolli, Aimee Selten, Celeste J M Koster, Daniëlle S C Maassen, Alba Montoro Canelo, Maureen Fredriks, Myrthe J A Koppers, Kim Clevers, Julia Klein, Vela Kaludjerovic, Jan Meeldijk, Emma W Pijnappel, Heggert G Rebel, Sven van Kempen, Sandra Crnko, Thijs Koorman, Aniello Federico, Francesco Valzano, Pieter Wesseling, Friso G J Calkoen, Jasper van der Lugt, Toine ten Broeke, Marcel Kool, Niels Bovenschen

**Affiliations:** Department of Pathology, University Medical Center Utrecht (UMCU), Utrecht, The Netherlands; Department of Pathology, University Medical Center Utrecht (UMCU), Utrecht, The Netherlands; Princess Máxima Center for Paediatric Oncology, Utrecht, The Netherlands; Department of Pathology, University Medical Center Utrecht (UMCU), Utrecht, The Netherlands; Department of Pathology, University Medical Center Utrecht (UMCU), Utrecht, The Netherlands; Department of Pathology, University Medical Center Utrecht (UMCU), Utrecht, The Netherlands; Department of Pathology, University Medical Center Utrecht (UMCU), Utrecht, The Netherlands; Department of Pathology, University Medical Center Utrecht (UMCU), Utrecht, The Netherlands; Department of Pathology, University Medical Center Utrecht (UMCU), Utrecht, The Netherlands; Department of Pathology, University Medical Center Utrecht (UMCU), Utrecht, The Netherlands; Department of Pathology, University Medical Center Utrecht (UMCU), Utrecht, The Netherlands; Department of Pathology, University Medical Center Utrecht (UMCU), Utrecht, The Netherlands; Department of Pathology, University Medical Center Utrecht (UMCU), Utrecht, The Netherlands; Department of Pathology, University Medical Center Utrecht (UMCU), Utrecht, The Netherlands; Center for Translational Immunology, University Medical Center Utrecht (UMCU), Utrecht, The Netherlands; Department of Pathology, University Medical Center Utrecht (UMCU), Utrecht, The Netherlands; Department of Pathology, University Medical Center Utrecht (UMCU), Utrecht, The Netherlands; Department of Pathology, University Medical Center Utrecht (UMCU), Utrecht, The Netherlands; Department of Pathology, University Medical Center Utrecht (UMCU), Utrecht, The Netherlands; Department of Pathology, University Medical Center Utrecht (UMCU), Utrecht, The Netherlands; Department of Pathology, University Medical Center Utrecht (UMCU), Utrecht, The Netherlands; Princess Máxima Center for Paediatric Oncology, Utrecht, The Netherlands; Princess Máxima Center for Paediatric Oncology, Utrecht, The Netherlands; Department of Neurosurgery, University Medical Center Utrecht (UMCU), Utrecht, The Netherlands; Department of Pathology, Amsterdam University Medical Center / VUmc, Amsterdam, The Netherlands; Princess Máxima Center for Paediatric Oncology, Utrecht, The Netherlands; Princess Máxima Center for Paediatric Oncology, Utrecht, The Netherlands; Department of Pathology, University Medical Center Utrecht (UMCU), Utrecht, The Netherlands; Division of Paediatric Neurooncology, German Cancer Research Center (DKFZ) and German Cancer Consortium (DKTK), Heidelberg, Germany; Hopp Children’s Cancer Center (KiTZ), Heidelberg, Germany; Princess Máxima Center for Paediatric Oncology, Utrecht, The Netherlands; Center for Translational Immunology, University Medical Center Utrecht (UMCU), Utrecht, The Netherlands; Department of Pathology, University Medical Center Utrecht (UMCU), Utrecht, The Netherlands

**Keywords:** *CD155*/*TIGIT*, immune checkpoints, medulloblastoma, organoids, *PD-L1*/*PD-1*

## Abstract

**Background:**

Medulloblastoma (MB) is one of the most prevalent pediatric brain malignancies and makes up approximately 20% of all primary brain tumors in children. Current treatment options are not curative for approximately 30% of patients and leave survivors with an impaired quality of life. Immune checkpoint inhibition can offer a novel targeted therapy but largely remains understudied in MB. The aim of this study was to determine whether dual immune checkpoint inhibition can be used as a novel targeted therapy in MB.

**Methods:**

We utilized single cell and single nuclei sequencing datasets of primary MB tumors, established Group 3 and Sonic Hedgehog MB cell lines and MB patient-derived xenograft (PDX) organoid models, and primary patient-derived MB tissue of all subtypes to study immune checkpoints and their blockade to target MB.

**Results:**

We identified the expression of immune checkpoint protein *CD155* on MB tumor cells and the expression of its inhibitory binding partner *TIGIT* on immune cells of MB patient-derived tissues, cell lines, and PDX MB organoids. In addition, while MB shows weak, if any, *PD-L1* protein expression, we found that MB cells can upregulate *PD-L1* expression upon stimulation by natural killer (NK) cells or interferon-γ as a putative immune evasive strategy. Subsequent immunotherapeutic interventions with FDA-approved antibodies Tiragolumab (anti-*TIGIT*), Durvalumab (anti-*PD-1*), and their combination potentiated primary NK cell activation and killing of MB cell lines and PDX-derived MB organoids.

**Conclusion:**

These data propose a translatable and novel immunotherapeutic strategy for children diagnosed with subgroups Sonic Hedgehog and Group 3 MB.

## Graphical Abstract

**Graphical Abstract:**
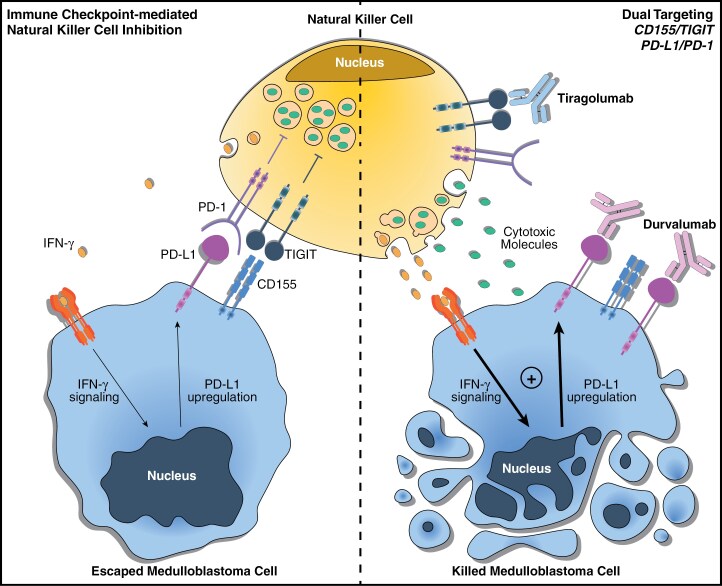

**Key Points**

- MB expresses the *CD155*-*TIGIT* immune checkpoint
- NK cells upregulate *PD-L1* on MB through interferon-γ
- Tiragolumab and Durvalumab potentiate NK cell-mediated killing of 3D MB organoids

**Importance of the Study**

Medulloblastoma (MB) is one of the most prevalent pediatric brain malignancies and makes up approximately 20% of all primary brain tumors in children. Current treatment options are not curative for 30% of patients and leaves survivors with a low quality of life. Immune checkpoint inhibition can offer a novel targeted therapy but remains largely understudied in MB. Here, we identified the expression of immune checkpoint proteins *CD155* (tumor cells) and *TIGIT* (immune cells) in medulloblastoma and proved that subsequent immunotherapeutic interventions with FDA-approved antibodies Tiragolumab (anti-*TIGIT*), Durvalumab (anti-*PD-1*), and their combination showed increased immune cell activation and enhanced killing of MB cell line and PDX-derived organoids. These data propose a translatable and novel immunotherapeutic strategy for patients diagnosed with subgroups Sonic Hedgehog and Group 3 MB and could also pave the way for similar treatments in other pediatric (brain) tumors.

Medulloblastoma (MB) is one of the most prevalent pediatric brain malignancies, reaching its peak incidence in children of 6 to 8 years old.^1^ Four main molecular groups are identified—Wingless (WNT), Sonic Hedgehog (SHH), Group 3, and Group 4—which can be further subdivided into distinct subtypes with different clinical and molecular characteristics.^2,3^ Current treatment options consist of surgical debulking, chemotherapy, and craniospinal radiation, resulting in a mean overall 5 year survival for all MB patients of approximately 70%, but survival strongly depends on the MB subgroup.^3^ Despite these multimodal treatment approaches approximately 30% of patients relapse, of which 95% eventually succumb.^4^ Furthermore, the treatment burden is extremely high resulting in complications such as growth disturbances, hormonal imbalances, mental issues, neurocognitive impairment, and multi-organ damage.^5^ Therefore, MB patients need more effective and less toxic therapies to improve survival and quality of life.

Immune checkpoints can inhibit appropriate cytotoxic immune responses in cancer.^6^ Therefore, immunotherapy (e.g. immune checkpoint inhibition [ICI], adoptive cell therapy, and dendritic vaccine therapy) started a new era in cancer treatment and has been extensively studied over the last two decades.^7^ Currently, ICI of anti-programmed cell death receptor-1 and its ligand-1 (*PD-1*/*PD-L1*) is mostly used and proven to be successful in multiple cancers.^8^ Through antibody-mediated blockade of these immune checkpoints, the immune system can be (re)activated to clear (brain) tumor cells.^9,10^ However, knowledge of immune checkpoint expression and functionality in MB patients is very limited. The most studied immune checkpoint *PD-L1* seems not to be expressed in MB, thereby rendering *PD-L1*/*PD-1* inhibition unsubstantial.^11–17^ Currently, no approved ICI treatment exists for MB.

*CD155* (also known as poliovirus receptor or *PVR*) is an immunoglobulin superfamily adhesion molecule involved in many physiological processes, such as cell adhesion, migration, and proliferation.^18^*CD155* has recently been recognized as an immune checkpoint due to its inhibitory capacity of cytotoxic immune cells.^19^ Several studies found that *CD155* is expressed in a variety of human malignancies.^20–24^ One of the binding partners of *CD155* is T cell immunoreceptor with immunoglobulin and ITIM domain (*TIGIT*), a receptor present on immune and natural killer (NK) cells.^19^*CD155*/*TIGIT* binding results in an inhibitory signal in the effector cell, thereby impairing its tumor-killing capacity. Tiragolumab is currently the first anti-*TIGIT* monoclonal antibody approved by the FDA and several new *TIGIT* blocking antibodies are currently being tested in vivo and in human trials^25^ in the context of melanoma,^26^ lung cancer,^27^ and liver cancer.^28^ However, for MB, the presence, functionality, and manipulation of the *CD155*/*TIGIT* pathway remains as yet unexplored.

In this study, we identified the expression of immune checkpoint proteins *TIGIT* and *CD155* in MB patient-derived tissues. In addition, we found that initially *PD-L1* negative MB cell lines and PDX-derived organoids can upregulate and use *PD-L1* as an immune evasive strategy following NK cell attack. Subsequent immunotherapeutic interventions with Tiragolumab (anti-*TIGIT*), Durvalumab (anti-*PD-1*), and their combination potentiate immune cell activation and enhances killing of MB cell lines and PDX-derived MB organoids.

## Materials and Methods

### Patient Tissue Samples

The study was performed under the tenets of the Helsinki Declaration (as revised in 2013) and all relevant national regulations and institutional policies. The study protocol was approved by the authors’ institutional review board (#PMCLAB2020.189). Collection of patient tissue samples was coordinated and approved by the institutional biobank (Princess Máxima Center for Pediatric Oncology, Utrecht, The Netherlands).

### Single Cell Sequencing Analysis

Single cell and single nuclei public datasets were obtained from GEO (GSE211362, GSE239854, GSE156053, GSE120974), or upon request (GSE253557) and re-analyzed to generate a general MB dataset. Analyses were performed using Seurat v5.1.0.^29–32^ Quality control metrics for nCounts and nFeatures were set as indicated in the original manuscripts. An additional quality metric was introduced to control high levels of ribosomal genes, considered as background noise. Cells with ribosomal genes content above 45% were excluded for further analysis. Data normalization was performed using SCT transformation^33,34^ regressing out from the analysis variables related to the content of ribosomal and mitochondrial genes. The top 3,000 variable features in the dataset were identified and then ribosomal genes were filtered out. The remaining variable genes were used for principal component analysis (PCA). Batch effect removal (or integration) was performed using Harmony^35^ and further Uniform Manifold Approximation and Projection (UMAP) dimensionality reduction was performed using the top 30 significant principal components. Shared nearest neighbor graph was performed with the first 30 principal components and cell clustering was achieved using the Louvain algorithm. Immune cell markers were identified based on the expression of the pan immune cell marker PTPRC (CD45). Myeloid and lymphoid lineages were subsetted into distinct data subsets and SCT transformation, PCA calculation and Harmony batch effect correction was re-calculated for each data subset. Differentially Expressed Gene analysis was performed on a per cluster level to obtain lists of cluster-specific genes used for cell annotation. Wilcoxon Rank Sum Test was used to highlight significant genes with Benjamini–Hochberg adjustment method and an adjusted *P* value threshold set on .05.

### Cell Culture

Cancer cell lines Daoy (*CD155*^+^ and *PD-L1*^+^ control),^36^ HD-MB03 (MB, Group 3 subgroup), and D283 (MB, Group 3 subgroup) were cultured in a 5% CO_2_ atmosphere at 37 °C.^37^

Daoy was cultured with Dulbecco’s Modified Eagle Medium (DMEM) (Gibco, cat. #31966-021) supplemented with 10% heat-inactivated fetal bovine serum (FBS) (Sigma, cat. #F7524), and 100 units penicillin and 100 µg/mL streptomycin (1% p/s) (Thermo Fisher, cat. #15140-122). HD-MB03 and YT-Indy (NK cell line) were maintained in RPMI 1640 medium (Gibco, cat. #72400-021), 10% heat-inactivated FBS, and 1% p/s. For HD-MB03, 20-25% conditioned medium, supernatant obtained from confluent HD-MB03 flasks that were spun down to pellet debris, was added to cultures. D283 was cultured in DMEM low glucose (Gibco, #31885-023) supplemented with 1% stable L-glutamine (200 mM, PAA, #M11-006), 20% heat-inactivated FBS, and 1% p/s. IL-2 independent NK-92 MI cells were maintained in MEM alpha no nucleosides (Gibco cat. #12561-056), with 10% heat-inactivated FBS, 10% heat-inactivated horse serum (Cytiva, cat. #SH300074.03), and 1% p/s.

### Primary NK Cells

Peripheral blood mononuclear cells were isolated from healthy human donors (UMC Utrecht, Mini Donor Dienst) by Ficoll-Paque (Cytiva, cat. #17144002) gradient centrifugation. Donors signed informed consent, according to the Declaration of Helsinki. The remaining erythrocytes were lysed on ice for 10 minutes in ammonium lysis buffer (10 nM KHCO_3_, 155 mM NH_4_Cl, 0.0037 mg/mL Na_2_EDTA, pH 7.4). Subsequently, negative selection NK isolation was performed using a magnetic cell separation system (MACS) with a human NK cell isolation kit (Miltenyi Biotec, cat. #130-092-657) and LS columns (Miltenyi Biotec, cat. #130-042-401), according to the manufacturer’s instructions. Populations of enriched primary NK cells typically contained 80% to 90% CD56^+^ cells, confirmed by flow cytometry (data not shown).

Before co-cultures, NK cells were activated in unsupplemented MB cell medium with 1000 IU/mL IL-2 (Miltenyi Biotec, cat. #130-097-742) and 50 ng/mL IL-15 (Miltenyi Biotec, cat. #130-095-765) for 2 hours at 37 °C.

### NK Cell—Medulloblastoma Cell Co-Culture

Daoy and MB cell lines were trypsinized (Trypsin/EDTA, Innoprot., cat. #0113), washed with PBS, and stained with 5 μM Cell Trace CFSE (Invitrogen, cat. C34554) in 10 mL unsupplemented medium for 20 minutes at 37 °C. After staining, cells were washed with supplemented medium, counted, and plated (Daoy: 6 × 10^4^ cells/mL, HD-MB03: 9 × 10^4^ cells/mL, and D283: 1.1 × 10^5^ cells/mL) overnight. Three wells were sacrificed and counted the next day to determine accurate effector: target (E:T) ratio. Then, effector cells were added either with or without 500 ng/mL total antibody treatment (Tiragolumab (Bio-Connect Life Sciences, Selleck Chemicals, cat. #A2028), Durvalumab (Imfinzi, Lot AAMX), and/or Purified Recombinant Human IgG1 Isotype Control (BioLegend, cat. #403502)). Co-culture was incubated for 48 hours and 5 hours at 37 °C for killing assay and NK cell activation assay, respectively.

After co-culture, killing assay cells were washed with PBS, harvested using 1% Trypsin/EDTA, and kept on ice. Samples were spun down for 5 minutes at 450 × *g* at 4 °C, resuspended in FACS buffer (PBS, 0.5% BSA, 0.1% NaN_3_), transferred to a V-bottom 96-well plate, and washed with FACS buffer before flow cytometry analysis. NK activation assay cells were transferred to a V-bottom 96-well plate directly and kept on ice until flow cytometry analysis.

### NK Cell—Medulloblastoma Cell *PD-L1* Priming Co-culture

MB cell lines were trypsinized, washed, stained, and plated as described above. Simultaneously, 10 ng/mL recombinant interferon- γ (IFN-γ, ImmunoTools, cat. #11343536) or recombinant tumor necrosis factor-α (TNF-α, ImmunoTools, cat. #11343015) was added to induce *PD-L1* expression. After 48 hours, cells were either subjected to *PD-L1* analysis as described below or the medium containing IFN-γ or TNF-α was washed off and three wells were counted to determine the accurate E:T ratio. Then, effector cells were added either with or without 500 ng/mL total antibody treatment (Tiragolumab, Durvalumab, and/or IgG1 Isotype Control) and co-culture was incubated for 6 h at 37 °C. After co-culture, cells were harvested and analyzed as described above.

### NK Cell—Medulloblastoma Organoid Co-culture

MB organoids MED113FH^38^ (patient-derived xenograft [PDX] primary SHH MB tumor) and B062-008 (PDX-derived recurrence SHH MB tumor) were gently pipetted and filtered through a 40 µm cell strainer to form a single-cell suspension. Then, cells were counted and plated (1 × 10^5^ cells/mL) and incubated for at least 24 hours at 37 °C. When 3D structured organoids had again formed (typically after 24-48 hours), three wells were sacrificed and counted to determine an accurate E:T ratio. Effector cells were added either with or without 500 ng/mL total antibody (Tiragolumab, Durvalumab, and/or Purified Recombinant Human IgG1 Isotype Control) and co-culture was incubated for 48 hours at 37 °C. After co-culture, cells were harvested and analyzed as described above.

### Flow Cytometry for Killing Assay

Cells were stained with 40 μL 7-AAD viability staining (1:40, BioLegend, cat. #420404) in FACS buffer for 20 minutes on ice in the dark. Then, cells were washed twice with FACS buffer, resuspended in 150 μL FACS buffer with Precision Count Beads (1:30, BioLegend, cat. #424902), and analyzed by Cytoflex (Beckman Coulter, cat. #C09753). Compensation was performed using CompBeads (BD Bioscience). Data was analyzed using CytExpert (Beckman Coulter, Inc., version 2.4.0.28).

### Flow Cytometry for Protein Expression

For extracellular surface markers, cells were harvested and directly stained with 40 μL of fluorescently tagged antibodies: anti-*PD-L1* (PE-Cyanine 7, 1:40, eBioscience, mouse IgG1κ, cat. #25-5983-42), anti-*CD155* (PE, 1:40, BioLegend, mouse IgG1κ, cat. #337610), anti-*TIGIT* (PE, 1:40, BioLegend, mouse IgG2aκ, cat. #372704), or the corresponding isotype controls (PE-Cyanine 7, eBioscience, mouse IgG1κ, clone P3.6.2.8.1; PE, BioLegend, mouse IgG1κ, cat. #400114; and PE, BioLegend, mouse IgG2aκ, cat. #400212).

For the NK activation assay for intracellular cytokines, cells were first fixed with 100 µl of 4% paraformaldehyde (PFA) in PBS for 30 minutes on ice in the dark. Cells were then washed with PBS and permeabilized using FACS buffer with 0.1% Saponin for 30 minutes on ice in the dark before intracellular staining with anti-IFN-γ (PE-Cyanine 7, 1:40, Becton and Dickinson, mouse IgG1κ, cat. #557844) or the corresponding isotype control (PE-Cyanine 7, eBioscience, mouse IgG1κ, clone P3.6.2.8.1).

### qPCR

Total RNA was isolated using TRIzol reagent (Thermo Fisher, cat. #15596026) and RNeasy Mini Kit (Qiagen, cat. #74104) according to the manufacturer’s protocol. cDNA was synthesized from total RNA using High-Capacity RNA to cDNA kit (Thermo Fisher, #4387406) and amplified using TaqMan Universal PCR Master Mix (Applied Biosystems, cat. #43-044-37). TaqMan gene expression assays for *GAPDH* (Hs02758991_g1), *CD155* (Hs00197846_m1), *TIGIT* (Hs00545087_m1), and *PD-L1* (Hs00204257_m1) were from Applied Biosystems. The real-time qPCR reactions were run on a ViiA 7 standard real-time PCR system (Applied Biosystems, cat. #4453534). Gene expression values were normalized against the *GAPDH* endogenous control. The relative gene expression ratios were obtained using the 2^-ΔΔCT^ method.

### Immunoblotting

Samples for immunoblotting were lysed in reducing Laemmli sample buffer (0.125 M Tris HCl pH = 6.8, 4% SDS, 20% glycerol, 0.004% bromophenol blue, 200 mM DTT), boiled for 5 minutes and stored at −20 °C until further use. Protein lysates were separated with 10% sodium dodecyl sulfate–polyacrylamide gel electrophoresis (SDS–PAGE) gels. Separated proteins were transferred to a methanol pre-activated polyvinylidene difluoride (PVDF) membrane (Immobilon®-P, cat #IPVH00010).

Immunoblotting continued by blocking membranes for 1 hour in blocking buffer (Tris-buffer saline (TBS) + 1% Tween (TBS-T), 5% nonfat dried milk (Nutricia, protifar)) at room temperature (RT). Primary antibodies—anti-*PD-L1* (1:1000, eBioscience, cat. #14-5983-82) and HRP-conjugated *GAPDH* antibody (1:1000, Santa Cruz Biotechnology, sc-47724; Lot #F2320)—were incubated overnight at 4 °C diluted in blocking buffer. Membranes were washed 3 × 10 minutes with blocking buffer at RT. Secondary HRP-conjugated goat anti-mouse antibody (1:5000, Jackson ImmunoResearch Laboratories, cat. #155-035-003) was diluted in blocking buffer and incubated for 1 hour at RT.

After incubation, membranes were washed with TBS and the signal was developed with ECL prime (Amersham cytiva, cat. #RPN2232). The signal was captured with a Chemi-Doc Imaging system from Bio-Rad.

### Immunohistochemistry

Tissue slides were deparaffinized, blocked, cooked in EDTA, and stained by primary antibodies against *PD-L1* (Roche, Ready-to-use (RTU) clone SP263, J17387), CD3 (Roche, RTU, 2GV6, K10044), and *CD155* (Cell Signaling clone D3G7H, 13544S, 1:500) was performed using an automated immunostainer (Benchmark Ultra, Ventana, Roche) using the OptiView DAB detection kit (REF: 760-700) and UltraVIEW Universal Alkaline Phosphatase Red Detection Kit (REF: 760-501). Immunohistochemistry for *TIGIT* (Abcam clone BLR047F, ab243903, 1:100) was performed manually (EDTA pH 9.0 was used as antigen retrieval step at 100 °C for 20 minutes, followed by 60 minutes of incubation with the primary antibody). Appropriate positive and negative controls were included in all stainings. For *PD-L1* detection, three consecutive slides were stained for CD3, *PD-L1*, and CD3 to be able to correlate CD3^+^ and *PD-L1*^+^ regions. After incubation with secondary antibody (Goat-anti-rabbit), slides were counterstained with hematoxylin, dried, and mounted. The presence of immune checkpoint proteins *CD155* and *TIGIT* was scored on reactivity (0, absent; C1, weak cytoplasmic; C2, moderate cytoplasmic; C3, intense cytoplasmic; M, membranous) and tumor cells positive (0% to 100%). Samples with reactivity ≥ C1 and tumor cells positive ≥ 10% were considered *CD155*^+^ or *TIGIT*^+^.

### Confocal Microscopy

Daoy, D283, and HD-MB03 cell lines were cultured on 10 µg/mL poly D Lysine hydrobromide (Sigma, cat. #p7405-5)-coated 10 mm diameter glass coverslips (Roth cat. #YX02.2) in 12-well plates in the earlier mentioned culture media. When cell density reached approximately 90% confluency, cells were cooled on ice and incubated with anti-*CD155* (BioLegend, mouse IgG1κ, cat. #337610, diluted 1:80 in medium to 0.6 µg/mL) or mouse isotype control (BioLegend, mouse IgG1κ, cat. #400114, diluted 1:320 in medium to 0.6 µg/mL) for 1 hour. After a triple wash with cold medium, all coverslips were incubated with secondary antibody goat anti-mouse-FITC (Sigma, cat. #F5387, 1:2500, to 1.5 µg/mL) for 45 minutes on ice. Coverslips were then gently washed three times with cold PBS supplemented with 0.90 mM CaCl_2_ and 0.49 mM MgCl_2_ and fixed with 1% PBS-buffered formaldehyde (Klinipath, cat. #4078-9001) for 10 minutes at RT. Cells were counterstained with 5 µg/mL 4’,6-diaminophenylindole (DAPI, Sigma D942), mounted in Mowiol 4-88 on object glasses (Waldemar Knittel, Starfrost 3 × 1 inch microscope slides) and kept in dark at 4 °C until confocal imaging.

Confocal microscopy was performed with a 405 nm and 488 nm-equipped laser-scanning microscope (LSM700 Zeiss). Imaging rendering was carried out with Imaris software. Emission bleed trough was minimized by multitracking.

### *TIGIT*/*CD155* Blockade Bioassay

*TIGIT*/*CD155* blockade bioassay (Promega, cat. #J2201) was performed according to the manufacturer’s instructions. To ensure consistent activation of the T cell receptor, 10 µl CD3/CD28 dimerization beads were added to the co-culture. HD-MB03 and D283 were included in the bioassay to study the role of *TIGIT*/*CD155* blockage in medulloblastoma cells. Anti-*TIGIT* (Promega, cat. #J2051) was used to block *TIGIT*/*CD155* interaction. After a 6-hour co-culture, bioluminescence was determined using a GloMax Discover microplate reader (Promega, GM3000).

### Three Dimensional Live Cell Imaging

B062-008 MB organoids were stained with CFSE CellTrace (Invitrogen ref. #C34554) and subsequently seeded with 30,000 cells per well in a black glass-bottom 96-well plate (Greiner bio-one cat. #96077307). Organoids were treated with 5 ng/mL IFN-γ (ImmunoTools cat. #11343536) and incubated for 48 hours. After incubation, primary NK cells were stained with CellTrace Violet (Invitrogen ref. #C34557A) and resuspended in RPMI 1640 medium with 2.5% BME (Cultrex cat. #3431-055-01), NucRed Dead 647 (Invitrogen ref. #R37113), and TO-PRO-3 (Invitrogen ref. #T3605) according to literature.^39^ Effector cells were added either with or without 500 ng/mL total antibody treatment (Tiragolumab, Durvalumab, and/or Purified Recombinant Human IgG1 Isotype Control) and imaged right away. Live cell imaging was performed using the Thunder Imager Live Cell on the DMi8 Leica microscope with z-stacks of 50 μm and continuous adaptive focus control. Images were taken every 30 minutes for 21.5 hours in total. The images were processed using large volume computational clearing Large Volume Computational Clearing (LVCC) to subtract background signal, and analysis was conducted using Imaris 10.0.0 software. After calculating the mean intensity signal per channel for each timepoint per z-stack, the dead cell ratio was determined according to the following formula:


$$\begin{array}{l} Dead\;cell\;ratio=\frac{\frac{\sum Red\;\;\;signal}{\sum Green\;\;\;signal}}{\sum Blue\;signal}=\frac{\frac{Dead\;\;\;organoids}{Total\;\;\;organoids}}{Total\;NK\;cells} \end{array}$$

To standardize the ratios, each z-stack’s ratio was normalized to the highest value found within that z-stack. Following this, the ratios were averaged per timepoint for the same experimental condition, which entailed averaging the two pictures in each well and accounting for any duplicates. To compare the 1:1 E:T ratios to the corresponding 0:1 E:T ratios, each 1:1 E:T ratio was divided by its corresponding 0:1 E:T ratio. Finally, the ratios were normalized against the highest value within their respective z-stacks. These computations were conducted using a custom R script. Final figures were constructed in GraphPad Prism software and presented as cumulative normalized dead cell ratio.

### RNA Microarray Analysis

The public dataset from Cavalli (Medulloblastoma; expression data; *n* = 763) was used for the expression analysis of *CD155*, *TIGIT*, and *PD-L1*^40^. Analysis was performed on ‘R2: Genomics Analysis and Visualization Platform (http://r2platform.com). Expression data is shown as 2log transformed data from raw values.

### Statistical Analysis

One-way ANOVA with Holm-Šidák’s multiple comparisons test and two-tailed paired *t*-test were performed in GraphPad Prism software (GraphPad Prism Software, LLC, version 9.3.0 (463)). *P*-values of less than 0.05 were considered to be significant. Statistical details of experiments are described in figure legends.

## Results

### Primary MB Show Abundant *CD155* Expression and *TIGIT*^+^ Immune Cell Infiltrate

**Figure 1.**
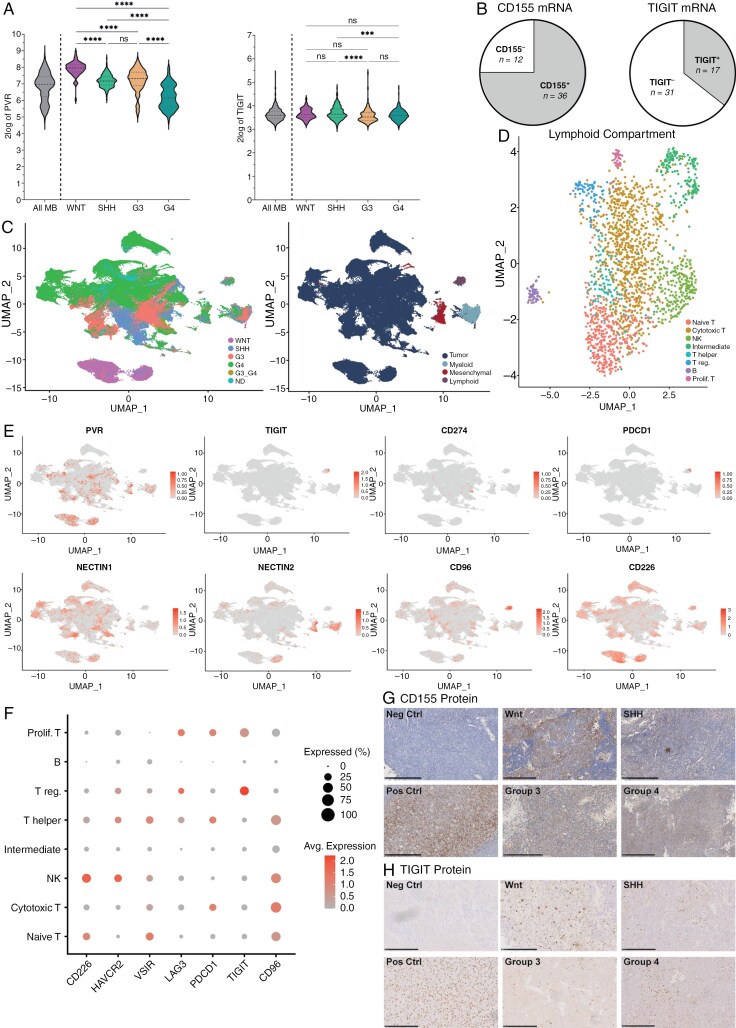
Primary MB show abundant *CD155* expression and *TIGIT* + immune cell infiltrate. (a) Online R2 genomics analysis and visualization platform was used for gene expression of *CD155* (*PVR,* left) and *TIGIT* (right). On the left of each graph the entire MB cohort (Cavalli, *n* = 763) is shown and on the right expression per MB subgroup is shown (WNT: *n* = 70, SHH: *n* = 223, G3: *n* = 144, G4: *n* = 326). Independent cohort of MB patients was analyzed for *CD155* mRNA (b, left) and protein (g) and *TIGIT* mRNA (b, right) and protein (h) (mRNA cohort *n* = 48, protein cohort *n* = 45). Single-cell transcriptomics clustering for molecular subtype (c, left) and cell type (c, right) was performed with subsequent analysis of the lymphoid compartment (d). Immune checkpoints protein mRNA expression was checked in (e) and investigated per cell type in (f). One-way ANOVA with Holm-Šidák’s multiple comparisons test (a) was used for statistical analysis; ** *P* < .01, *** *P* < .001, **** *P* < .0001. Statistical analysis was performed automatically in the R2 platform. (g,h) Scale bars represent 250 µm. Abbreviations: G3, Group 3; G4, Group 4; MB, Medulloblastoma; Neg Ctrl, Negative control (medulloblastoma tissue); Pos Ctrl, Positive control (tonsil tissue); SHH, Sonic hedgehog; WNT, Wingless.

The presence and functionality of the immune checkpoint *CD155*/*TIGIT* axis remains unexplored in MB. To address this gap, we sought to determine its expression in mRNA microarray data of 763 MB patients (http://r2platform.com).^40^ Expression of *CD155* (gene name: *PVR*) was higher compared to *TIGIT* (Figure 1a). For *CD155*, the expression pattern differed significantly between subgroups with MB-WNT tumors showing highest expression and MB-G4 tumors showing lowest (Figure 1a, left). For *TIGIT*, only slight changes in expression pattern were observed between subgroups (Figure 1a, right). Subsequently, mRNA expression was measured in our independent tissue cohort of 48 MB patients (patient characteristics in Table 1) of which 36 and 17 cases were found to be *CD155*^+^ and *TIGIT*^+^ (Figure 1b), respectively. To further elucidate these expression patterns, single-cell transcriptomics of MB tumors from published datasets were utilized to identify distinct clusters (Figure 1c), with a particular focus on the myeloid and lymphoid clusters—key players in immune checkpoint blockade. The myeloid compartment was enriched with immunosuppressive tumor-associated macrophages (TAMs), monocytes, dendritic cells, and activated TAMs (Supplementary Figure S1a), consistent with previous findings.^41,42^ In agreement with Vermeulen et al. (2018), who showed that T and NK cells were dispersed over the tumor tissue, our single cell sequencing data show that T cells were the most abundant lymphoid population, while NK cells were sparsely represented (Figure 1d). Both *CD155* and *TIGIT* mRNA were detected and differentially expressed across MB subgroups (Figure 1e), along with mRNA of other interacting proteins such as *NECTIN1*, *NECTIN2*, *CD96*, and *CD226* and well-known immune checkpoint *PD-L1*/*PD-1* (Figure 1e). Interestingly, the myeloid compartment exhibited minimal expression of immune checkpoint ligands of *CD155* (Supplementary Figure S1b). In contrast, lymphoid cells demonstrated significant expression of *TIGIT*, *PD-1* (gene name: PDCD1), CD226, and CD96. Regulatory T cells (T regs) emerged as key contributors to the suppressive tumor microenvironment, with the highest average mRNA expression of the inhibitory molecule *TIGIT* (Figure 1f). To confirm protein expression, tissues from 45 MB patients were subjected to immunohistochemistry analysis of *CD155* (Figure 1g) and *TIGIT* (Figure 1h). *CD155* was expressed differentially within and between all MB subgroups with most prevalent expression in MB-WNT (Table 2). Infiltrating immune cells that express *TIGIT* were found in 93.3% of all MB cases (Table 2). Thus, these data show abundant *CD155* mRNA and protein expression in primary MB tissues. Furthermore, *TIGIT*^+^ cells are present in the large majority of MB cases.

**Table 1.** Patient characteristics.

		*n* or value	%
**Gender**	Male	28	58.3
	Female	15	31.3
	Unknown	5	10.4
**Age (years)**	Mean ± SD (*n*)	8.3 ± 4.7 (43)	
	Range	0.4 to 17.8	
	Unknown	5	
**Histological type**	Classic	30	65.2
	Desmoplastic nodular	9	19.6
	Extensive nodular	1	2.2
	Anaplastic	2	4.3
	Unknown	6	13.0
**Molecular classification**	WNT	5	10.9
	SHH	10	21.7
	Group 3	11	23.9
	Group 4	16	34.8
	Unknown	6	13.0

Abbreviations: SHH, sonic hedgehog; WNT, wingless.

**Table 2.** *CD155* and TIGIT protein expression per MB subgroup.

Molecular classification	Total	WNT	SHH	Group 3	Group 4	Unknown
Total cases (*n*)	48	5	10	11	16	6
*CD155* ^+^ cases (*n*)	23	5	5	8	2	3
*CD155* ^+^ cases (%)	47.9	100.0	50.0	72.7	12.5	50.0
*TIGIT* ^+^ cases (*n*)	44	5	9	11	14	5
*TIGIT* ^+^ cases (%)	91.7	100.0	90.0	100.0	87.5	83.3
Histological type	Total	Classic	Desmoplastic nodular	Extensive nodular	Anaplastic	Unknown
Total cases (*n*)	48	30	9	1	2	6
*CD155* ^+^ cases (*n*)	23	14	5	0	2	2
*CD155* ^+^ cases (%)	47.9	46.7	55.6	0.0	100.0	33.3
*TIGIT* ^+^ cases (*n*)	44	27	9	1	2	5
*TIGIT* ^+^ cases (%)	91.7	90.0	100.0	100.0	100.0	83.3

Abbreviations: SHH, sonic hedgehog; WNT, wingless.

### *CD155*/*TIGIT* Blockade Potentiates NK Cell-Mediated Killing of MB Cells

**Figure 2.**
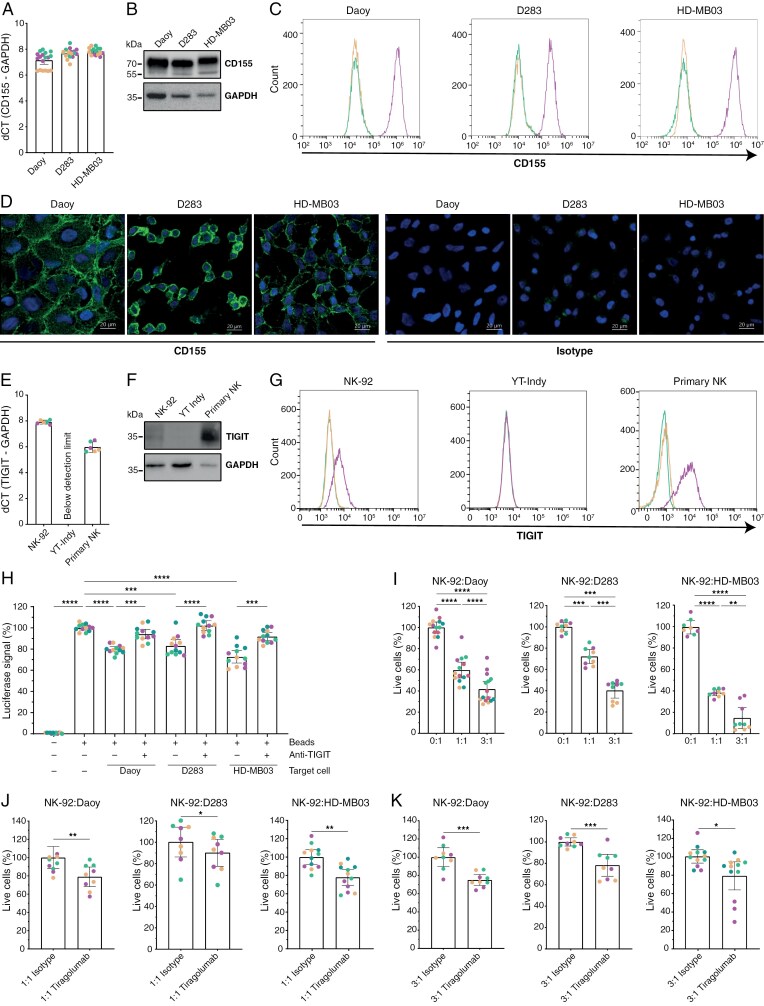
*CD155*/*TIGIT* blockade potentiates NK cell-mediated killing of MB cells. Baseline mRNA and protein expression levels of *CD155* were examined for MB cell lines by qPCR (a), western blot (b), flow cytometry (c), and confocal microscopy (d); all *n* = 3. For NK cells, baseline mRNA and protein expression levels of *TIGIT* were examined by qPCR (e), western blot (f), and flow cytometry (g); all *n* = 3. In flow cytometry plots, green = unstained, yellow = isotype, and purple = anti-*CD155* (c) or anti-*TIGIT* (g). (h) TCR-transduced Jurkat cells were cultured and TCR was activated by adding CD3/CD28 activation beads (+/- ‘Beads’). MB cells were added with or without anti-*TIGIT* to examine reduction and recovery in TCR activation, respectively; *n* = 4. (i) Baseline killing of Daoy (control, left) and MB cell lines D283 (middle) and HD-MB03 (right) by NK-92 in different E:T ratios; all *n* ≥ 3. (j,k) Effect of Tiragolumab treatment on the killing of Daoy (left) and MB cell lines D283 (middle), and HD-MB03 (right) by NK-92 at E:T 1:1 (j) and 2:1 (k); *n* ≥ 3. For (a,e,h–k), data is shown as mean ± 95% confidence interval (CI), and different colors correspond to multiple biological replicates. One-way ANOVA with Holm-Šidák’s multiple comparisons test (h–i) and two-tailed paired *t*-test (j,k) were used for statistical analysis, * *P* < .05, ** *P* < .01, *** *P* < .001, **** *P* < .0001. Abbreviation: dCT, delta CT.

**Figure 3.**
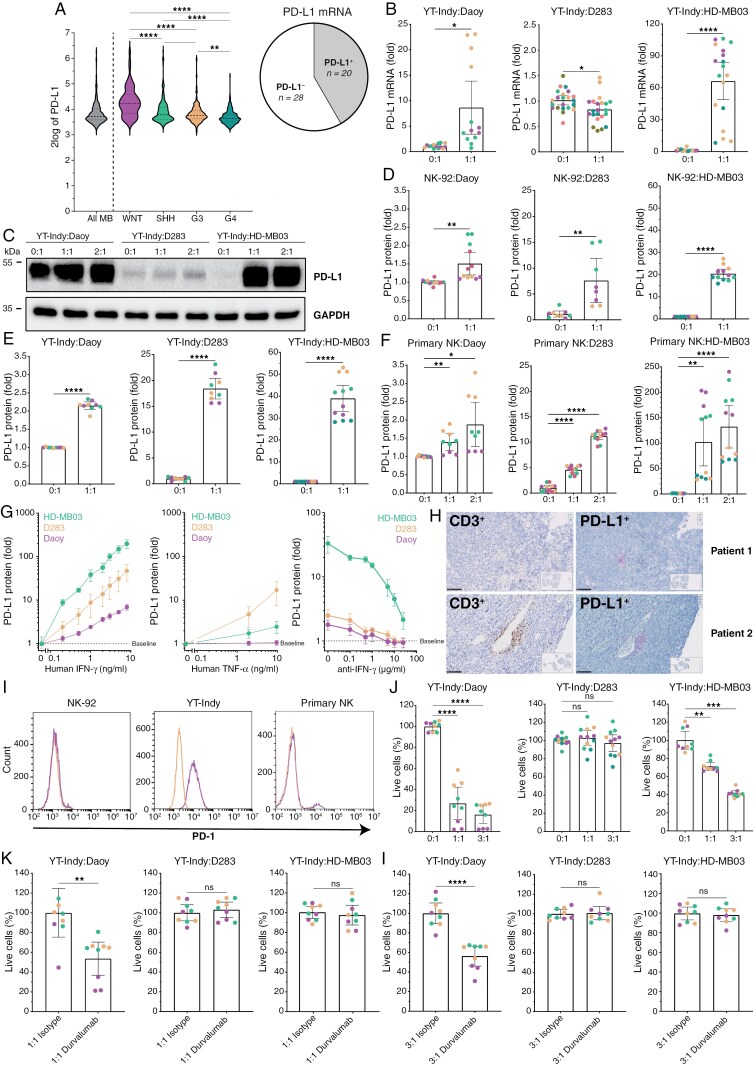
NK cells induce *PD-L1* expression via IFN-γ and *PD-L1*/*PD-1* blockade potentiates NK cell-mediated killing of MB cells. (a, left) Online R2 genomics analysis and visualization platform for RNA sequencing was used for gene expression of *PD-L1*. On the left of the graph the entire MB cohort (*n* = 763) is shown and on the right expression per MB subgroup is shown (WNT: *n* = 70, SHH: *n* = 223, G3: *n* = 144, G4: *n* = 326). Independent cohort of MB patients was analyzed for (a, right) *PD-L1* mRNA (*n* = 48). (b) *PD-L1* mRNA upregulation after co-culture between YT-Indy and Daoy (left), D283 (middle), and HD-MB03 (right); all *n* = 3. (c) *PD-L1* protein upregulation after co-culture between YT-Indy and MB cell lines; a representative example of *n* = 3. (d–f) *PD-L1* protein upregulation on flow cytometry after co-cultures with NK-92 (d), YT-Indy (e), and primary NK (f) and Daoy (left), D283 (middle), and HD-MB03 (right); all *n* ≥ 3. (g) *PD-L1* protein upregulation on flow cytometry after administration of different concentrations IFN-γ (left) or TNF-α (middle) or after co-culture with YT-Indy and anti-IFN-γ (right) for Daoy (purple), D283 (yellow), and HD-MB03 (green); all *n* = 3. (h) CD3 (brown) and *PD-L1* (purple) staining on back-to-back MB patient-derived tissue slides, *n* = 2. Scale bars represent 100 µm. (i) Baseline protein expression level of *PD-1* on NK-92, YT-Indy, and primary NK cells by flow cytometry. In flow cytometry plots, yellow = isotype, and purple = anti-*PD-L1*. (j) Baseline killing of Daoy (left) and MB cell lines D283 (middle), and HD-MB03 (right) by YT-Indy in different E:T ratios; all *n* ≥ 3. (k,l) Durvalumab treatment on the killing of Daoy (left) and MB cell lines D283 (middle), and HD-MB03 (right) by YT-Indy at E:T is 1:1 (k) and3:1 (l); all *n* ≥ 3. For (b,d–f,j–l), data is shown as mean ± 95% confidence interval (CI), and different colors correspond to multiple biological replicates. One-way ANOVA with Holm-Šidák’s multiple comparisons test (f,j) and two-tailed paired *t*-test (b,d,e,k,l) were used for statistical analysis, * *P* < .05, ** *P* < .01, *** *P* < .001, **** *P* < .0001. Abbreviation: IFN-γ, Interferon-γ; TNF-α, Tumor necrosis factor-α.

To investigate the functional role of immune checkpoint *CD155*/*TIGIT* in MB, we employed several representative MB cell lines and NK cells. *CD155* was expressed on Daoy and all available MB cell lines (D283—Group 3, HD-MB03—Group 3; Figure 2a–d), and *TIGIT* expression was confirmed on NK-92 and primary NK cells, but not on YT-Indy NK cells, as determined by qPCR, immunoblot, flow cytometry and immune fluorescence (Figure 2e–g). The functionality of the *CD155*/*TIGIT* interaction was assessed by an anti-*TIGIT* blockade assay using T cell receptor (TCR)-transduced reporter Jurkat cells and MB cells. All MB cell lines inhibited TCR activation, as measured by luciferase reporter assays, which could be fully rescued by adding an anti-*TIGIT* blocking antibody (Figure 2h). To further investigate the functional role of *TIGIT*, NK-92 cells were co-cultured with MB cells in different E(ffector):T(arget) ratios. Dose-dependent (E:T) baseline killing was observed in all MB cell lines (Figure 2i). Subsequent blockade of *CD155*/*TIGIT* immune checkpoint with anti-*TIGIT* blocking antibody Tiragolumab enhanced the killing capacity of NK-92 cells towards all MB cell lines with all tested E:T ratios of 1:1 (Figure 2j) and 3:1 (Figure 2k). Taken together, these data show that *CD155*/*TIGIT* blockade with anti-*TIGIT* Tiragolumab potentiates NK cell-mediated killing of MB-Group 3 cells.

### NK Cells Induce *PD-L1* Expression via IFN-γ and *PD-L1*/*PD-1* Blockade Potentiates NK Cell-Mediated Killing of MB Cells

MB expresses very low levels, if any, of *PD-L1*, rendering anti-*PD-1*/*PD-L1* treatment not suitable for MB tumors.^17,43^ However, mRNA levels of *PD-L1* could be detected in the RNA microarray database of 763 MB patients (http://r2platform.com)^40^ and showed highest expression in MB-WNT subgroup (Figure 3a, left). Also in the independent cohort (Table 1), *PD-L1* mRNA was detected in 43% (17 out of 40) of all cases (Figure 3a, right). If *PD-L1* protein expression in MB is detected, it exposes in focal areas in less than 2% of the tumor.^43^ Therefore, we investigated whether NK cells can (locally) upregulate tumor *PD-L1* in MB. Indeed, after challenging MB cells with NK cells, *PD-L1* mRNA increased in Daoy and HD-MB03 (up to 200-fold), but not in D283 cells (Figure 3b) and *PD-L1* protein levels increased in all MB cell lines as determined by immunoblot (Figure 3c). Flow cytometry confirmed this *PD-L1* protein upregulation on the cell surface of all tested MB cell lines after co-culturing with NK-92 (Figure 3d), YT-Indy (Figure 3e), and primary NK cells (Figure 3f). Mechanistically, we investigated whether IFN-γ or TNF-α can upregulate *PD-L1*. Recombinant human IFN-γ (Figure 3g, left) or TNF-α (Figure 3g, middle) was added to MB cells and subsequently led to *PD-L1* protein upregulation in all tested MB cell lines in a dose-dependent manner. Additionally, *PD-L1* upregulation could be abolished by adding anti-IFN-γ blocking antibody to a co-culture of NK and MB cells (Figure 3g, right), indicating that IFN-γ is the most dominant cytokine involved. Finally, in primary MB patient-derived tissue, positive *PD-L1* staining was confirmed in focal areas, mostly, but not exclusively, in perivascular regions that also showed high CD3^+^ immune infiltrates (Figure 3h). To examine if *PD-L1* upregulation impairs NK-cell mediated killing, we first confirmed *PD-1* protein expression on YT-Indy and primary NK cells, but not on NK-92 (Figure 3i) and looked at baseline killing of MB cells by YT-Indy (Figure 3j). Blockade of *PD-L1*/*PD-1* immune checkpoint with Durvalumab resulted in 50% extra killing capacity by NK cells in Daoy but not D283 and HD-MB03 cell lines (Fig 3k-l). Collectively, these data show that at least NK cells upregulate *PD-L1* expression in MB cells via IFN-γ and that subsequent *PD-L1*/*PD-1* blockage with Durvalumab increases NK cell killing capacity of Daoy but not MB-Group 3 cells.

### Dual Targeting of *CD155*/*TIGIT* and *PD-L1*/*PD-1* Enhance Primary NK Cell Activation and Killing Towards MB Cells and Organoids

**Figure 4.**
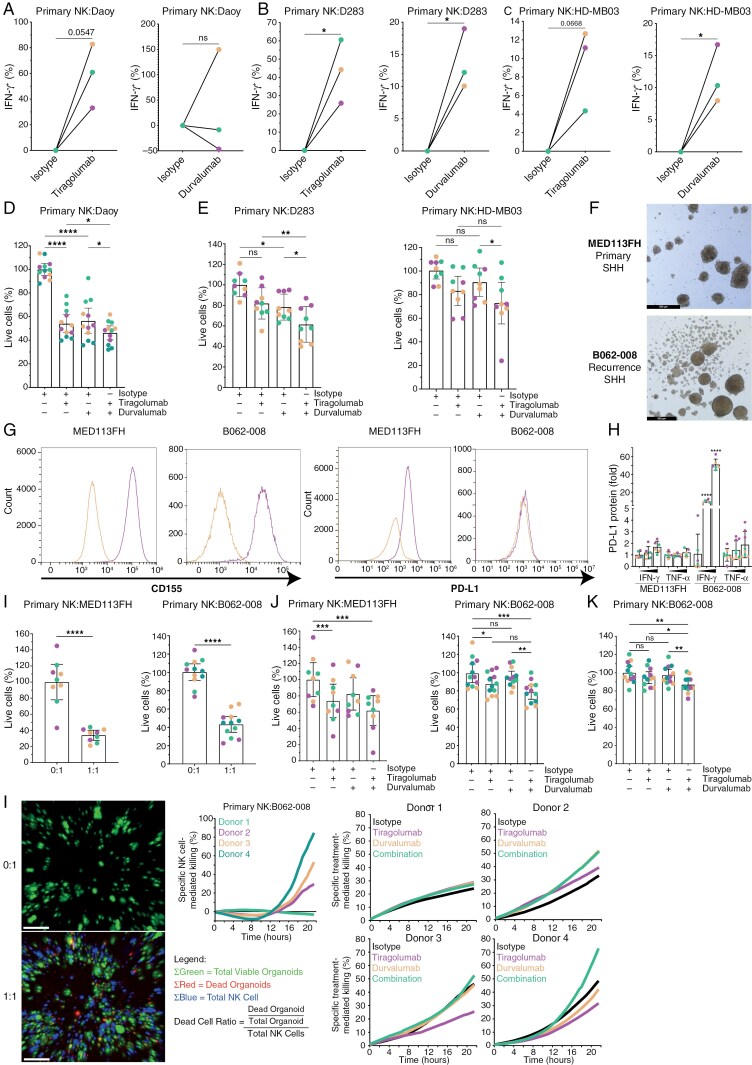
Dual targeting of *CD155*/*TIGIT* and *PD-L1*/*PD-1* enhance primary NK cell activation and killing towards MB cells and organoids. Therapy of Tiragolumab (left) and Durvalumab (right) compared to isotype increases IFN-γ^+^ primary NK cells in Daoy (a), D283 (b), and HD-MB03 (c) co-cultures, all *n* ≥ 3. Combination therapy of Tiragolumab and Durvalumab on Daoy (d) in E:T = 2:1 and IFN-γ primed D283 (e, left), and HD-MB03 (e, right) in E:T = 1:1, all *n* ≥ 3. (f) Microscopic image of 3D organoid structure in culture. Scale bars represent 500 μm. (g) Baseline expression levels of *CD155* (left) and *PD-L1* (right), both *n* = 3. In flow cytometry plots, yellow = isotype and purple = anti-*CD155* or anti-*PD-L1*. (h) IFN-γ- and TNF-α-induced upregulation of *PD-L1* expression (*n* = 3) and (i) baseline killing of MED113FH (left) and B062-008 (right) by primary NK cells, *n* = 4. Combination therapy of Tiragolumab and Durvalumab in an E:T ratio of 1:1 in MED113FH (j, left) and B062-008 (j, right and k) without (j) and with (k) priming by IFN-γ to induce *PD-L1* expression, all *n* = 4. (l, left) Representative examples of real-time imaging screenshots from co-cultures of primary NK cells and B062-008 organoids taken every four hours. Green = alive B062-008 organoids, red = dead B062-008 organoids, blue = primary NK cells, white bars represent 100 µm, *n* = 4. (l, middle) Baseline killing of B062-008 by primary NK cells measured by real-time imaging using cumulative cell death analysis, *n* = 4. (l, right) Donor-specific effects on single and combination treatment of Tiragolumab and Durvalumab measured by real-time imaging using cumulative cell death analysis, *n* = 4. Data (d,e,h–k) is shown as mean ± 95% confidence interval (CI). For (a–c,l), different colors correspond to different NK cell donors, and for (d,e,h–k) different colors correspond to multiple biological replicates. One-way ANOVA with Holm-Šidák’s multiple comparisons test (d,e,h,j,k) and two-tailed paired *t*-test (a–c,i) were used for statistical analysis, * *P* < .05, ** *P* < .01, *** *P* < .001, **** *P* < .0001. Abbreviation: IFN-γ, Interferon-γ; TNF-α, Tumor necrosis factor-α.

Dual treatments co-targeting *TIGIT* and *PD-L1* have been proposed in recent literature in the context of colorectal cancer and lung squamous cell carcinoma.^44,45^ We examined NK cell activation status in co-cultures of MB cells with primary NK cells from healthy donors. More IFN-γ^+^ NK cells appeared after treatment with either Tiragolumab or Durvalumab compared to isotype control (Figure 4a-c). The killing capacity of primary NK cells towards *PD-L1*^+^ Daoy cells enhanced after a single treatment with either Tiragolumab or Durvalumab (Figure 4d). Moreover, combination treatment showed better killing compared to single treatment (Figure 4d). For D283 and HD-MB03 cells, *PD-L1* was first upregulated through pre-incubating MB cells with IFN-γ after which the killing capacity of primary NK cells was evaluated. In both these MB cell lines, the combination of Tiragolumab and Durvalumab significantly improved primary NK cell-mediated killing compared to single treatments (Figure 4e). Next, we sought to translate our findings to a primary (MED113FH) and recurrent (B062-008) PDX-derived MB organoid model of a which high-resolution light microscopic images (Figure 4f) are displayed. Both MB organoid models have been classified by DNA methylation as SHH. *CD155* protein expression was confirmed on both MED113FH and B062-008 cell membrane (Figure 4g, left) whereas *PD-L1* protein expression was limited (Figure 4g, right), but could be induced after incubation with IFN-γ in a dose-dependent manner (Figure 4h). Baseline killing of MED113FH (Figure 4i, left) and B062-008 (Figure 4i, right) organoids by primary NK cells was confirmed, whereafter in all cases, combination treatment of Tiragolumab and Durvalumab was more effective than single treatment (Figure 4j-k). Subsequently, real-time analysis of B062-008 death induced by primary NK cells was performed (Figure 4l). Three out of four primary NK cell donors were capable of achieving baseline killing (Figure 4l), albeit with different efficiencies. Also treatment-mediated killing was donor-specific, but, in all cases, the combination of Tiragolumab and Durvalumab resulted in more primary NK cell-mediated B062-008 killing compared to isotype treatment (Figure 4l). In conclusion, dual targeting of *CD155*/*TIGIT* and *PD-L1*/*PD-1* immune checkpoints potentiates primary NK cell-mediated cytotoxicity in MB cells and PDX-derived primary and recurrent MB organoids.

## Discussion

In this study we show that *CD155* immune checkpoint is expressed in all MB subgroups and that interfering with the *CD155*/*TIGIT* axis using anti-*TIGIT* Tiragolumab potentiates MB tumor cell killing by NK cells (Figs. 1 and 2). Furthermore, while MB shows weak, if any, *PD-L1* protein expression, we found that *PD-L1* mRNA and protein expression on MB can be induced by NK cells at least through IFN-γ release (Figure 3). In line with these data, we demonstrate that dual treatment with anti-*TIGIT* Tiragolumab and anti-*PD-L1* Durvalumab potentiates primary NK cell activity and anti-tumor cytotoxicity towards MB cell lines and PDX MB organoids (Figure 4). Thus, our data propose a novel and quickly translatable immunotherapeutic strategy that may counteract (pediatric) MB.

Over 70 clinical trials employing anti-*TIGIT* therapy are currently ongoing,^46^ of which most are investigating dual targeting of checkpoint inhibitors in combination with the standard of care treatment. Why mono ICI treatment yields mixed results remains unclear. This might be related to donor differences in immune cells and/or cancer cells. Alternatively, shared intracellular signal transduction pathways (redundancy and back-up) require dual checkpoint blockade to enhance NK cell-mediated killing of MB cells. Currently, combination therapy of anti-*TIGIT* and anti-*PD-L1* is being researched as a treatment option for cancers such as lung cancer,^47^ glioblastoma, and neuroblastoma.^48,49^ In the CITYSCAPE phase II clinical trial, Tiragolumab was combined with anti-*PD-L1* treatment in *PD-L1* positive non-small cell lung carcinoma patients.^47^ Dual targeting with Tiragolumab and Atezolizumab showed promising results in liver cancer additive to standard care.^28^ Our data also strongly suggest to combine anti-*TIGIT* with anti-*PD-L1* therapy to boost NK cell efficiency in MB.^50,51^

MB has very low, if any, protein expression of *PD-L1* rendering the *PD-L1*/*PD-1* axis probably not suitable as ICI target.^17,52^ However, Martin et al. (2018) found that IFN-γ and radiation can be used to induce *PD-L1* expression in MB cell lines. In addition, we now show that NK cells can induce *PD-L1* protein expression most efficiently through IFN-γ secretion after two days and that *PD-L1* protein can be detected on primary MB tissue. This expression is highest to perivascular regions coinciding with high immune infiltrates.^53^ A possible explanation for low *PD-L1* expression in MB is that immune cells are suppressed by the immune suppressive tumor microenvironment^54^ and thus unable to produce and/or secrete IFN-γ to induce *PD-L1* upregulation. Our data suggest that if immune infiltrates in the tumor are re-activated and/or enhanced, *PD-L1* rebecomes a potential tumor defence mechanism and thus an immunotherapeutic target in MB. Hence, several *PD-L1* negative tumors were found to respond to anti-*PD-L1*/*PD-1* therapy through an unexplained mechanism, resulting in FDA approval of these checkpoint inhibitors for *PD-L1* negative malignancies.^55^ This could indicate *PD-L1* induction might not be necessary to maintain treatment efficacy. These notions open the possibility that *PD-L1*/*PD-1* targeting in MB can work, either as ICI alone or together with strategies to overcome the immune suppressive microenvironment. Currently, two clinical trials (NCT03173950 and NCT04730349) are recruiting and investigating anti-*PD-1* treatment in MB tumors.

One obstacle for antibody treatment of MB (depending on subtype) is the blood-brain-barrier, which prevents large proteins to pass to the brain. However, research indicates that antibody treatment can still be effective in the cerebrospinal fluid if administrated intravenously.^56^ New approaches are investigated to enhance antibody transfer and efficacy in the central nervous system^57^ such as using ultrasound to open the blood-brain-barrier.^58^ Alternatively, as surgery is still used as standard care, an Ommaya reservoir can be placed, rendering the possibility to directly inject drugs intracranially.^57^

MB tumors are heterogeneous in that the amount of immune infiltrates largely varies among patients.^17^ ICI could be beneficial for MB tumors that contain substantial levels of immune infiltrates, as immune-cold tumors seem to respond less.^59^ For MB patients lacking infiltrating cytotoxic lymphocytes, ICI treatment can be combined with cellular infusions (e.g. CAR-T cells or NK cells) of which the efficiency remains to be studied in vivo.^45^

In conclusion, we show that dual blockade of *CD155*/*TIGIT* and *PD-L1*/*PD-1* immune checkpoints with Tiragolumab and Durvalumab significantly increases primary NK cell-mediated killing of MB cells and organoids. Further research is necessary for the potential clinical use of these agents to treat MB patients.

## Supplementary Material

vdaf099_suppl_Supplementary_Figure_S1

## Data Availability

Additional and supporting data is available upon reasonable request.
