# Supplementary material for: Dual targeting of CD155/TIGIT and PD-L1/PD-1 immune checkpoints potentiates NK cell-mediated cytotoxicity in medulloblastoma
Source: Neurooncol Adv. 2025 May 18;7(1):vdaf099. doi: 10.1093/noajnl/vdaf099 (PMC12284645; doi:10.1093/noajnl/vdaf099)
Supplement: vdaf099_suppl_Supplementary_Figure_S1 [file vdaf099_suppl_supplementary_figure_s1.docx]

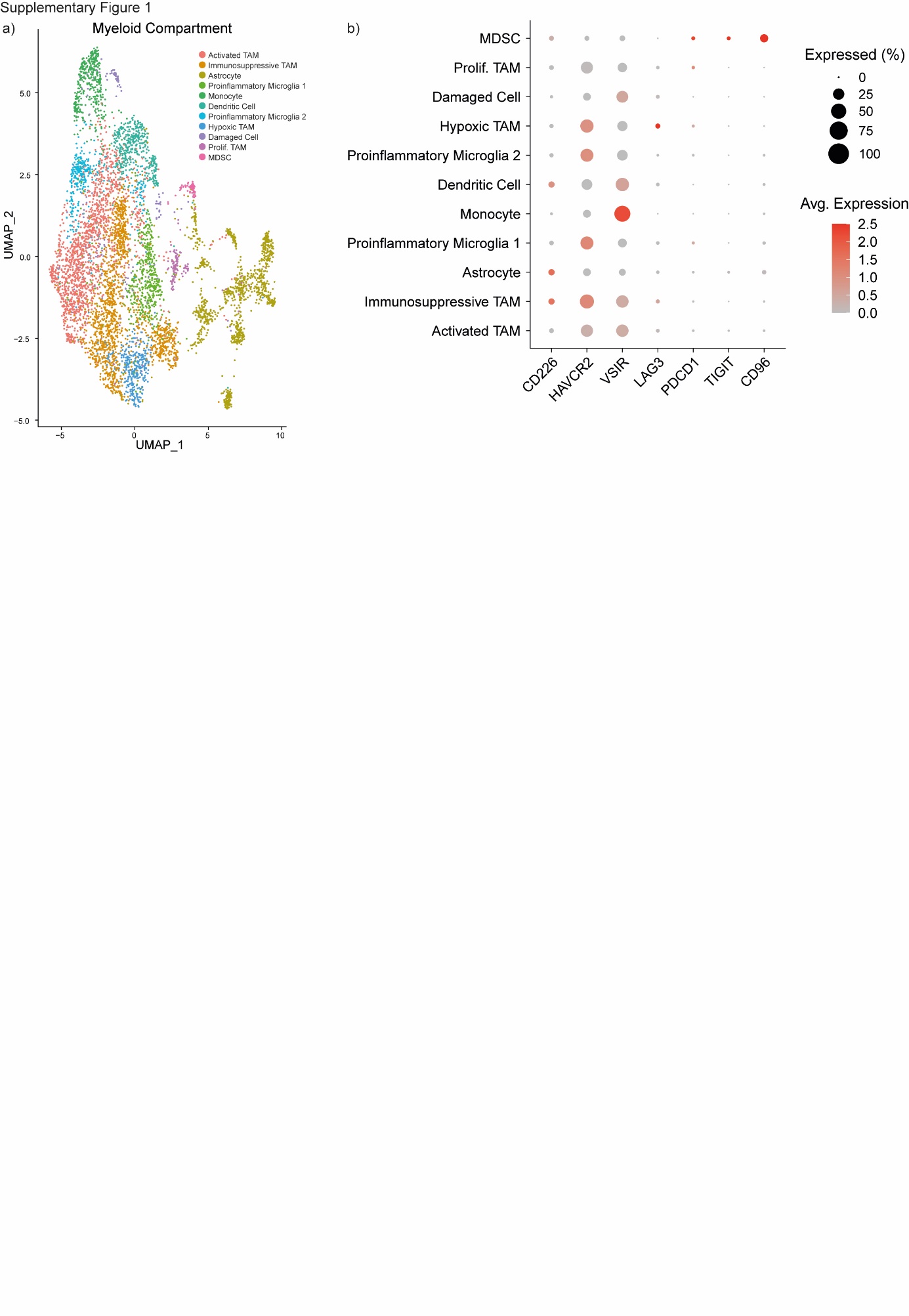


Supplementary Figure 1 – Myeloid compartment analysis of primary MB.

**(a)** Single-cell transcriptomics analysis of the lymphoid compartment with **(b)** subsequent analysis of immune checkpoint proteins per cell type. Abbreviation: MDSC, Myeloid-derived suppressor cell; TAM, Tumour-associated macrophage.
